# iRGD-mediated MPN functionalization of MSNs for targeted delivery of MIF: a potential strategy for placenta accreta spectrum therapy

**DOI:** 10.3389/fbioe.2025.1684943

**Published:** 2025-11-21

**Authors:** Fanying Zeng, Peng Chen, Longxia Tong, Guolin He

**Affiliations:** 1 Laboratory of the Key Perinatal Diseases, Key Laboratory of Birth Defects and Related Diseases of Women and Children, Ministry of Education, Chengdu, Sichuan, China; 2 Department of Obstetrics and Gynecology, West China Second University Hospital, Sichuan University, Chengdu, Sichuan, China; 3 Hi-Tech Zone Hospital for Women and Children, West China Second University Hospital, Sichuan University, Chengdu, Sichuan, China; 4 Department of Gynecology and Obstetrics, Meishan Women and Children’S Hospital, Meishan, China

**Keywords:** placenta accreta spectrum, mesoporous silica nanoparticles, metal–phenolic networks, internalization RGD peptide, drug delivery and sustained release

## Abstract

Placenta accreta spectrum (PAS) is a severe pregnancy complication characterized by abnormal placental invasion, leading to life-threatening hemorrhage during delivery. Current management strategies face challenges between the systemic side effects of pharmacological interventions and the risks of invasive surgery. This study develops a targeted nanotherapeutic platform using iRGD peptide-functionalized mesoporous silica nanoparticles (MSNs) coated with metal–phenolic networks (MPNs) for mifepristone (MIF) delivery. The MPN-coated MSNs exhibited enhanced drug-loading capacity (275 μg/mg) and sustained-release profiles (83.3% release over 48 h). *In vitro* studies demonstrated excellent biocompatibility and selective uptake in trophoblast cells via αvβ3 integrin targeting. In a pregnant mouse model, the internalization RGD peptide (iRGD)-modified nanoparticles showed preferential placental accumulation and induced significant abortion through targeted trophoblast apoptosis, as evidenced by reduced chorionic gonadotropin levels and histological analysis. Although this proof-of-concept study demonstrates a promising targeted therapeutic strategy using healthy animal models, we acknowledge the limitation of not using true PAS pathological models. Our findings establish a foundation for developing precision nanomedicines for placental disorders, with future studies required to validate efficacy in disease-specific models.

## 1 Introduction

Placenta accreta spectrum (PAS), a severe pregnancy complication, is characterized by the abnormal adherence of the placenta, leading to its invasion beyond the uterine lining into the myometrium or even penetrating the uterine wall into adjacent organs, which often results in the inability of the placenta to detach normally during delivery, causing severe hemorrhage and threatening maternal life (Ghosh et al., 2023; Jauniaux et al., 2019). Pathologically, PAS is classified into placenta accreta, placenta increta, and placenta percreta, based on the depth of chorionic villi invasion into the myometrium. PAS is induced by multiple factors, involving endometrial damage from previous surgeries such as cesarean sections and abnormal differentiation of trophoblast cells, which is a primary cause of excessive invasion of the placenta into the uterine wall (Luke et al., 1966). The incidence of PAS has significantly increased from 0.02% in the 1970s to approximately 0.17% currently, partly due to the changes in risk factors such as the increasing rate of cesarean sections (Yu and Leung, 2021; Miller et al., 1997; Betran et al., 2021; Givens et al., 2022).

Retained products of conception (RPOC) complicated by PAS is a critical obstetric condition with two primary therapeutic approaches: pharmacological management and surgical intervention. Pharmacological treatment, a conservative strategy, aims to stimulate uterine contractions or induce necrosis of residual trophoblastic tissue through hormonal agents to promote the expulsion of retained tissue; however, these drugs often carry systemic side effects. Surgical methods, while achieving complete removal of retained placental tissue, are associated with high procedural risks, elevated costs, and irreversible damage to uterine function and fertility (Grover et al., 2020; Tol et al., 2019; Bartels et al., 2021). To reduce surgical risks and preserve fertility, there has been an improvement toward less invasive and drug-based conservative treatments (Sentilhes et al., 2023). However, these treatments still face challenges such as immunosuppression, allergic reactions, and systemic side effects. There is an urgent need for safer and more effective therapeutic approaches, particularly those that can target the affected tissues more precisely to minimize side effects. As a result, based on the increasing incidence of PAS and the limitations of current treatment options, the development of an advanced strategy for PAS is particularly urgent.

Mesoporous silica nanoparticles (MSNs), known for their excellent specific surface area, tunable pore sizes, and good biocompatibility, have emerged as promising drug delivery vehicles (Zhang et al., 2022; Croissant et al., 2017; Zhu et al., 2019; Author anonymous, 1989). These nanoparticles can encapsulate a high payload of drugs and can be surface-modified to enhance their targeting capabilities, thereby improving the specificity and efficacy of treatments (Lohiya and Katti, 2022; Giannakoudakis et al., 2021; Chen et al., 2019; Mura et al., 2013). In cancer therapy, for instance, MSNs modified with targeting ligands such as antibodies or peptides can selectively accumulate in tumor tissues, enhancing drug concentration at the site of action while minimizing systemic toxicity (Lohiya and Katti, 2022; Liu et al., 2020). For the precise and potent intervention required in PAS, MSNs are not only an alternative but also a superior platform. Their high loading capacity, excellent stability, and unparalleled versatility for surface functionalization and controlled release make them ideally suited to overcome the key challenges of placental drug delivery (Xu et al., 2023). Recent advancements in biomaterials have also highlighted the potential of plant polyphenols, one category of natural compounds known for their low cost, renewability, and excellent biocompatibility (Author anonymous, 1989; Wei and Haag, 2015). These compounds can form metal–phenolic networks (MPNs) through coordination with metal ions, providing a novel approach for surface modification and functionalization of nanoparticles (Tang et al., 2017; Park et al., 2014; Ju et al., 2022; Blanco et al., 2015; Gao et al., 2014). MPNs can also be tailored to enhance the stability, controlled release, and targeted delivery of drugs, offering a versatile platform for improving drug delivery systems.

**FIGURE 1:**
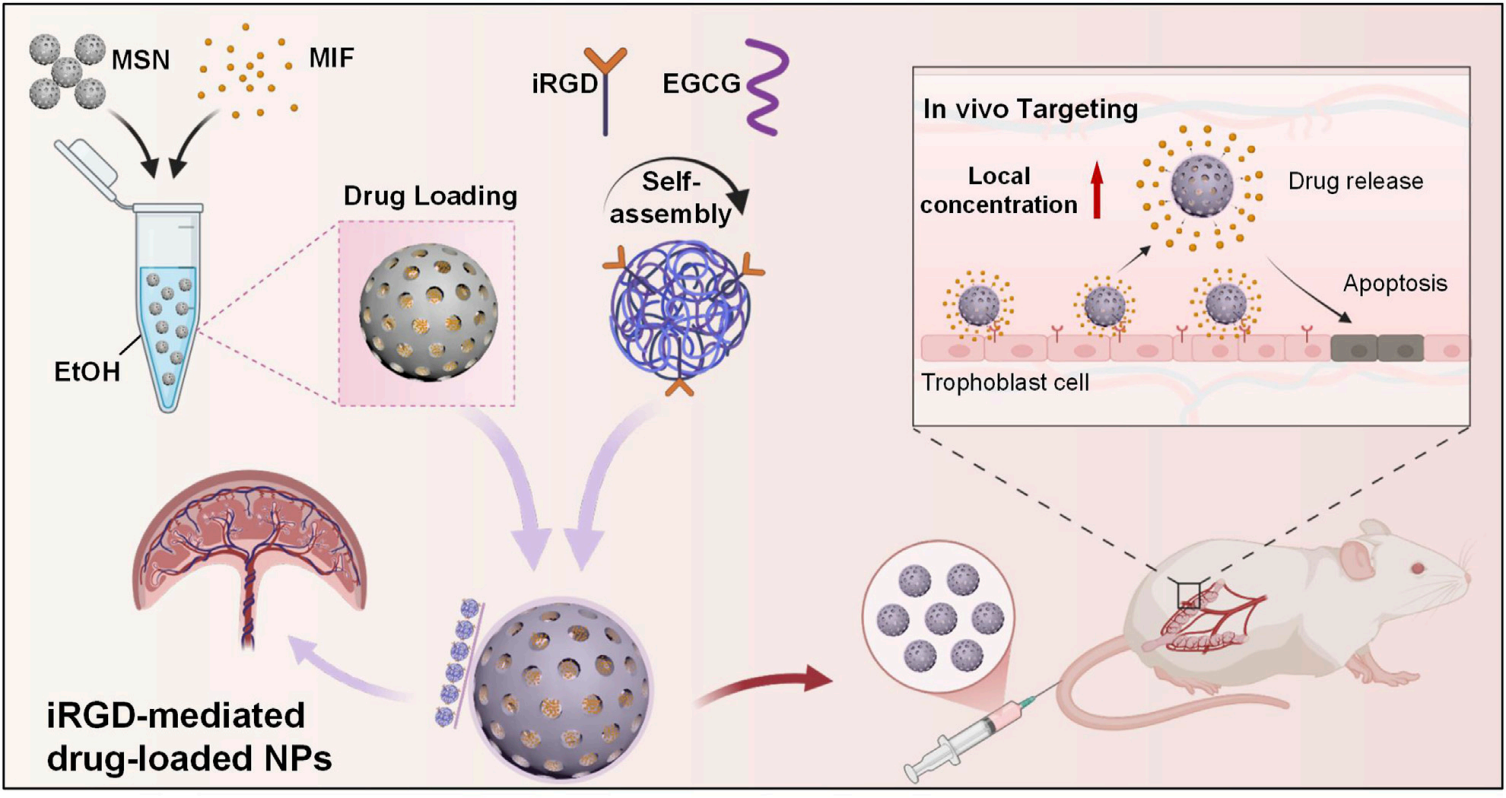
Scheme of the targeted drug delivery system for PAS treatment using MSNs encapsulated with MIF.

In this study, we explored a novel targeted drug delivery system for PAS treatment using MSNs encapsulated with mifepristone (MIF), a drug capable of inhibiting progesterone generation and used for PSA treatment, functionalized with an internalization RGD peptide (iRGD), which has potential in targeting placental tissues by binding to αvβ3 integrin, a receptor significantly expressed in invasive trophoblast cells (Tang et al., 2024; Li et al., 2022). As shown in Figure 1, by combining the unique properties of MSNs and the targeting capabilities of iRGD, we can potentially enhance the therapeutic index of mifepristone, ensuring that the drug is delivered directly to the site of placental invasion while minimizing exposure to healthy tissues. The proof-of-concept study represents a significant step forward in the treatment of PAS, offering a targeted and minimally invasive therapeutic option, and future studies are required to validate efficacy in PAS pathological models.

## 2 Methods and materials

### 2.1 Materials

Analytical grade (AR) epigallocatechin gallate (EGCG), ZnSO_4_, KBr, and MIF were purchased from Sigma-Aldrich (St. Louis, MO, United States). MSN was purchased from XFNANO (Cat: 102888, Jiangsu, China). Dulbecco’s modified Eagle medium: F12 (DMEM/F12), Roswell Park Memorial Institute 1640 medium, 1× phosphate-buffered saline (PBS), 100× penicillin–streptomycin, and trypsin (without EDTA) were obtained from Thermo Fisher Scientific (Waltham, MA, United States). Fetal bovine serum (FBS) was sourced from HyClone (Suzhou, China). iRGD was obtained from MedChemExpress (United States). HSA-FITC and HSA-cy7 were purchased from QiyueBio (Xi’an, China). BALB/c mice were procured from Envigo Biotechnology Co., Ltd. (Chengdu, China).

### 2.2 Methods

#### 2.2.1 Diameters and zeta potential detection

A PBS solution of MSNs (1 mg/mL) with a total volume of 1 mL was prepared. The solution was ultrasonicated in an ultrasonic cleaner at 100% frequency for 5 min to ensure thorough dispersion. Then, the mesoporous MSNs solution was transferred to the Malvern ZETA potential sample cell. After standing for 3 min at 25 °C, the particle size distribution was measured, and a plot was generated using the Zetasizer Nano ZSP (Malvern, United Kingdom).

#### 2.2.2 MPN assembly and iRGD conjugation

The MPN coating was constructed by sequentially adding 500 μL of EGCG solution (10 mg/mL) to 500 μL of MSN suspension (5 mg/mL) under vortex mixing for 60 s to facilitate uniform adsorption of EGCG onto the MSN surface. The mixture was centrifuged at 100 × g for 3 min, followed by three washes with 1× PBS to remove unbound components. The pellet was resuspended in 1 mL of PBS, and 10 μL of ZnSO_4_ solution (10 mg/mL) was introduced under 30 s of vortexing to initiate coordination between EGCG and Zn^2+^ ions, resulting in the formation of a stable MPN network on the MSN surface. For iRGD functionalization, 50 μL of iRGD solution (10 mg/mL) was premixed with 500 μL of EGCG solution via 60 s of vortexing to form EGCG–iRGD complexes prior to MSN addition, with subsequent steps identical to the standard MPN assembly procedure.

#### 2.2.3 Fourier-transform infrared spectrometer detection

A 1 mL EGCG-Zn^2+^-based MPN aqueous solution, MSN dispersion, and MPN-modified MSN dispersion (MPN@MSN) were prepared and frozen at −80 °C for 3 h. Then, the samples were freeze-dried for 12 h using an f SCIENTZ-18N Freeze-dryer (Scientz, Shanghai). Afterward, 2 mg of the sample was taken and mixed with 200 mg of KBr. Fourier-transform infrared (FT-IR) spectra were recorded using a Fourier-transform infrared spectrometer (Thermo Fisher Scientific, Nicolet 6700, United States), with a resolution of 4 cm^-1^ in the range of 4,000–400 cm^-1^.

#### 2.2.4 UV–VIS spectrophotometer detection

A 1 mL EGCG-Zn^2+^-based MPN aqueous solution (MPN group), MSN dispersion (MSN group), and MPN-modified MSN dispersion (MPN@MSN group) were prepared, respectively. After transferring the solutions to quartz cuvettes, the absorption curves of each group were scanned over the full wavelength range (200–800 nm) using a UV–visible spectrophotometer (Youke, Shanghai) under a slit width of 2 nm.

#### 2.2.5 Scanning electron microscope and energy-dispersive spectrometer detection

For scanning electron microscopy (SEM) (JEOL, JSM-7500F, Japan) and energy-dispersive spectrometry (EDS) tests, MSN and MPN@MSN dispersions were prepared independently. In particular, 10 μL of each dispersion was carefully pipetted onto a silicon wafer with dimensions approximately 5 × 5 mm^2^. Subsequently, the samples were allowed to air-dry naturally at room temperature. In the MPN@MSN group, during the imaging process, EDS tests were simultaneously conducted to analyze the elemental distribution (in particular, C, N, O, and Zn) on the surface of the MSNs.

#### 2.2.6 Transmission electron microscopy detection

For transmission electron microscopy (TEM) characterization, MSN and MPN@MSN dispersion were prepared. Subsequently, 10 μL of this dispersion was carefully pipetted onto a copper grid pre-coated with a carbon film. After complete natural drying, the sample was examined using a JEM-1400-FLASH (JEOL) operating at an accelerating voltage of 200 kV.

#### 2.2.7 Drug loading and release assay

Dimethyl sulfoxide (DMSO), anhydrous ethanol (EtOH), methanol (MT), and ethyl acetate (EAC) were used to explore the loading efficiency of MSNs for MIF. Five milligrams of MIF were accurately weighed and dissolved in each solvent to prepare a 1 mg/mL solution. Subsequently, 5 mg of MSNs were added to the MIF solution (5 mg/mL) in a tube and shaken at 100 rpm on a shaker. Samples were collected at 0.5, 1, 2, 4, 8, 12, 24, and 48 h, and the unloaded MIF supernatant was obtained by centrifugation. Drug-loading efficiency was recorded and calculated using a micro-volume spectrophotometer (NanoDrop 3000, Thermo Fisher Scientific).

For the *in vitro* drug release study on MPN@MSN, a 3 mL dispersion of MPN@MSN at 1 mg/mL was prepared in 1 × PBS. The dispersion was placed on a shaker at 37 °C and 100 rpm, and samples were collected at 0.5, 1, 2, 4, 8, 12, 24, and 48 h. After centrifugation, the supernatant was measured using a micro-volume spectrophotometer (NanoDrop 3000, Thermo Fisher Scientific).

#### 2.2.8 Cell culture

NIH/3T3 cells were cultured in DMEM, while KGN and HTR-8/SVneo cells were cultured in RPMI 1640. All culture media were supplemented with 10% fetal bovine serum (FBS) and 1% penicillin–streptomycin, and the cells were incubated at 37 °C with 5% CO_2_.

#### 2.2.9 CCK-8 assay

NIH/3T3 cells in the logarithmic growth phase were seeded at 5 × 10^3^ cells/well in a 96-well plate. Groups included control, MSNs (10, 50, 100, 250, and 500 μg/mL), EGCG (10 μM), and MPN@MSN (10, 50, 100, 250, and 500 μg/mL). After cell adhesion, the medium was replaced with fresh medium plus corresponding reagents. Cells were incubated for 24 h and washed three times with sterile 1 × PBS, and then 100 μL of fresh medium and 10 μL of CCK-8 reagent were added. After 4 h of incubation, absorbance at 450 nm was measured. The calculation formula is as follows:

$$\text{Cell viability}=\frac{OD_{\text{sample}}‐OD_{\text{medium}}}{OD_{\text{control}}‐OD_{\text{medium}}}\times 100\%.$$

#### 2.2.10 Apoptosis assay

Apoptosis in NIH/3T3 cells was evaluated using the Annexin V–FITC/propidium iodide (PI) kit. In brief, 1 × 10^5^ cells/well were seeded in a 6-well plate and cultured in 2 mL of untreated DMEM for 12 h. After being treated as above, the cells were trypsinized (EDTA-free) and washed three times with 1 × PBS. Finally, apoptosis of NIH/3T3 cells was detected according to the manufacturer’s instructions using a flow cytometer (Dakewe, Beijing, China).

#### 2.2.11 Live and dead cell staining assay

NIH/3T3 cells cultured in different groups were gently washed three times with 1 × PBS, taking care to avoid the removal of dead cells. Cells were then digested with trypsin, collected into 1.5 mL EP tubes, and centrifuged at 1,000 rpm for 5 min; the supernatant was discarded. Cells were resuspended in a 1:1,000 diluted mixture of calcein-AM and PI staining solutions (KeygenBio, Jiangsu), incubated for 30 min. After incubation, cells were centrifuged at 1,000 rpm for 5 min, washed with 1 × PBS, and resuspended. Finally, 50 μL of stained cells were placed on a glass slide and observed under a fluorescence microscope (Olympus, Japan).

#### 2.2.12 Cell targeting and phagocytosis assay

Cells were seeded into confocal dishes and cultured until reaching 70%–80% confluence. FITC-labeled MPN@MSN–iRGD nanoparticles were then added to the medium, followed by incubation for 0.5, 1, or 2 h. At each time point, cells were washed twice with 1 × PBS to remove residual nanoparticles. LysoTracker Red (Beyotime, Shanghai) was applied according to the manufacturer’s protocol to label lysosomes, with 1 h incubation under light-protected conditions. After replacing the staining solution with fresh medium, intracellular fluorescence signals were captured using laser scanning confocal microscopy (Leica, Germany).

#### 2.2.13 *In vivo* targeting assay

On day 14 post-conception, BALB/c mice were injected via the tail vein with 100 μL of Cy7-iRGD-labeled MPN@MSN dispersion. After 30 min, the mice were deeply anesthetized with 5% isoflurane. Then, euthanasia was performed by cervical dislocation, which was confirmed by the cessation of breathing and the absence of a pedal reflex. Subsequently, the animals were dissected. The uterus and major organs were arranged on a blackboard and imaged using an *in vivo* imaging system (IVIS Spectrum, PE, United States).

#### 2.2.14 TUNEL apoptosis assay

After treatment, the TUNEL assay was performed to assess apoptotic cells in the frozen sections of mouse placentas. Placental tissue sections were first fixed with 4% paraformaldehyde for 15 min at room temperature. Following fixation, sections were permeabilized with 0.2% Triton X-100 in PBS for 10 min. The TUNEL assay was then carried out using the Colorimetric TUNEL Apoptosis Assay Kit (Beyotime, Shanghai), following the manufacturer’s instructions. Apoptotic cells were visualized by green fluorescence. DAPI staining was applied to identify cell nuclei, and sections were mounted with an anti-fade mounting medium to preserve fluorescence. To further characterize trophoblastic cells, CK7 was detected using a primary antibody against CK7 (1:200, Proteintech, Wuhan), followed by incubation with a red fluorescent secondary antibody. Images were captured using a confocal microscope to observe the localization of apoptotic cells and CK7-positive cells.

#### 2.2.15 Mouse chorionic gonadotropin ELISA detection

Blood samples were collected from pregnant mice in different treatment groups via the retro-orbital route. The samples were immediately centrifuged at 3,000 × g for 10 min at 4 °C to separate the serum. The serum was then stored at −80 °C until analysis. Chorionic gonadotropin (CG) levels in the serum were quantified using a Mice Chorionic Gonadotropin (CG) ELISA Kit (QiyiBio, Shanghai), following the manufacturer’s instructions. Absorbance was measured at 450 nm using a microplate reader, and CG concentrations were determined based on a standard curve generated from known concentrations of CG standards. All measurements were performed in duplicate to ensure accuracy and reproducibility.

#### 2.2.16 Alanine aminotransferase (GPT/ALT) activity assay

Blood samples were collected from pregnant mice in different treatment groups. Serum was separated by centrifugation at 4 °C and stored at −80 °C until analysis. The activity of glutamic–pyruvic transaminase (GPT) in serum was determined using a Glutamic–Pyruvic Transaminase Activity Assay Kit (Solarbio, Beijing), according to the manufacturer’s instructions. In brief, 20 µL of serum was added to each well of a 96-well microplate. The reaction mixture, containing substrate, coenzyme, and buffer, was added to each well and incubated at 37 °C for 30 min. Absorbance was measured at 340 nm using a microplate reader. The ALT activity was calculated based on the standard curve and expressed in units per liter (U/L). All assays were performed in triplicate.

#### 2.2.17 Hemolysis assay

Hemocompatibility was evaluated through a standardized hemolysis assay using fresh whole blood collected from BALB/c mice via orbital venous plexus puncture and anticoagulated with heparin. Erythrocytes were isolated by centrifugation at 2,000 × g for 10 min and washed with PBS until the supernatant became clear. A 2% (v/v) erythrocyte suspension was prepared and incubated with MSN, MM, or iMSN@MM nanoparticles for 3 h at 37 °C with gentle shaking. Ultrapure water (H_2_O) and PBS were used as positive and negative controls, respectively. After incubation, samples were centrifuged, and the absorbance of the supernatant was measured at 540 nm.

#### 2.2.18 H&E staining

Placental and organ specimens were fixed in 4% paraformaldehyde for 24 h to preserve native morphology. Sequential dehydration was performed using a graded ethanol series, with 45 min of incubation at each concentration. Tissues were cleared in xylene and embedded in paraffin within molds, positioning the cutting surface downward. Paraffin blocks were sectioned using a microtome (5 μm thickness), and sections were air-dried prior to baking at 65 °C for 1 h, followed by extended baking (2 h). For dewaxing, sections underwent two 10-min xylene immersions, followed by rehydration through graded ethanol and washing with 1 × PBS. Hematoxylin and eosin (H&E) staining was conducted by sequential immersion in hematoxylin for 10 min, followed by differentiation rinses, eosin counterstaining for 5 min, and final dehydration. Sections were cover-slipped with neutral resin mounting medium under bubble-free conditions. Digital imaging was performed using a Pannoramic MIDI Digital Pathology System (3D-HISTECH, Hungary).

#### 2.2.19 Statistical analysis

All experiments were independently repeated in triplicate to ensure reproducibility. Data are presented as the mean ± standard deviation (SD). Statistical analyses were performed using SPSS 24.0 (IBM, United States). Intergroup comparisons were conducted using Student’s t-test (two groups), Fisher’s exact test (rate data), repeated-measures ANOVA, or Dunnett’s/LSD tests (three or more groups). Graphical representations were generated using GraphPad Prism 8.0 (GraphPad Software, United States). Statistical significance was denoted as follows: *p ≤ 0.05, **p ≤ 0.01, ***p ≤ 0.001, and ****p ≤ 0.0001.

## 3 Results and discussion

### 3.1 Characterization of MPN-coated MSNs

**FIGURE 2:**
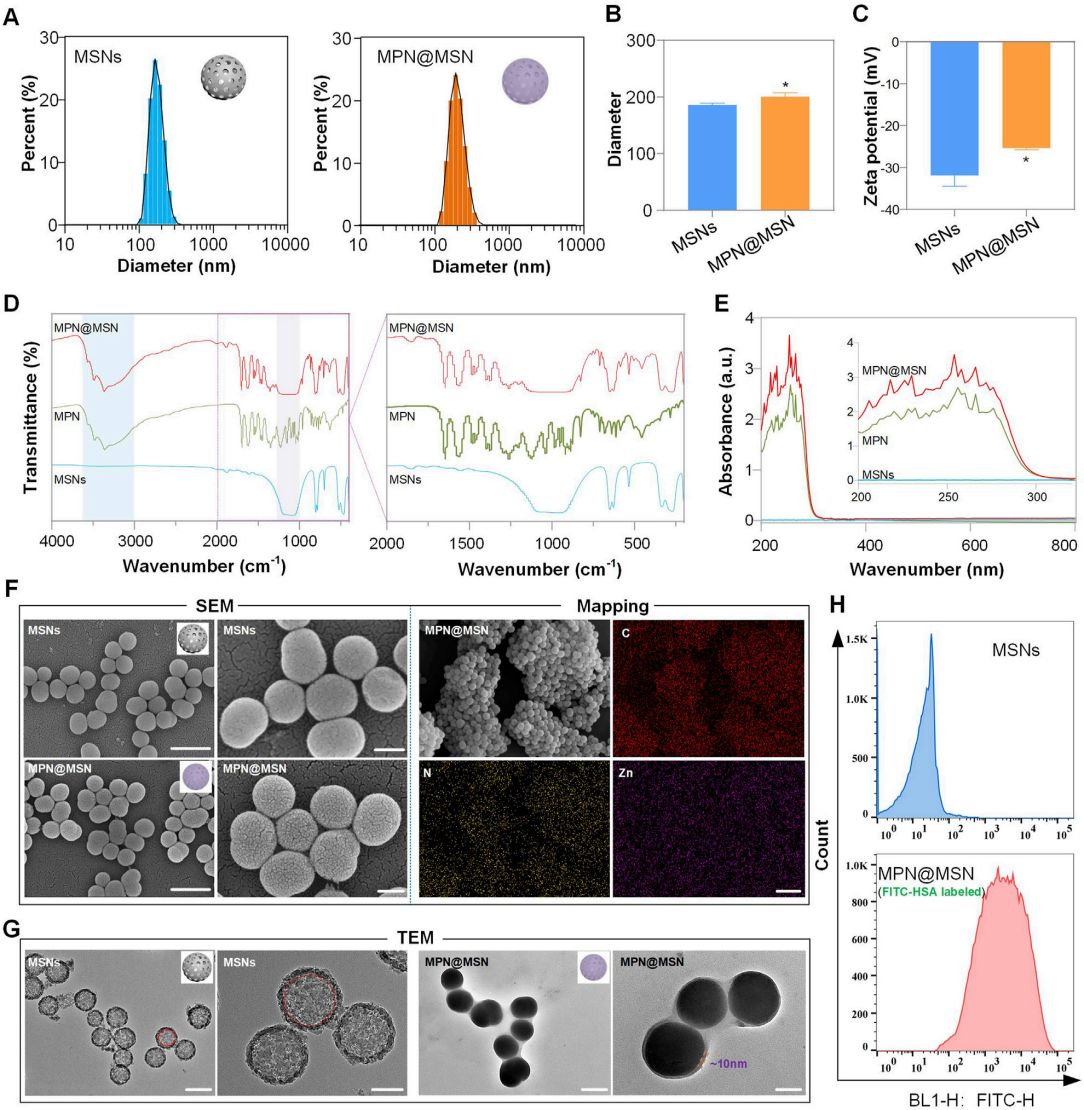
Characterization of MPN-coated MSNs. **(A–C)** Size distribution **(A,B)** and zeta potential **(C)** of MSNs before and after MPN modification. **(D)** FT-IR spectra showing characteristic peaks of EGCG–Zn coordination and Si–O–Si bonds (1,100 cm^-1^). **(E)** UV–VIS spectra demonstrating blue shift after MPN coating. **(F)** SEM images revealing an increase in surface roughness post-modification. Scale bars: 500 nm (left) and 200 nm (right). In addition, EDS elemental mapping confirming Zn distribution (purple) on MPN@MSN. **(G)** TEM images showing a 10-nm MPN layer (yellow arrows) on MSNs. Scale bars: 500 nm (left) and 100 nm (right). **(H)** Fluorescence intensity distribution of MSNs and MPN@MSN (FITC-HSA labeled). Data are represented as mean ± SD (n = 3). *p < 0.05.

The particle size of nanoparticles is closely related to their metabolic rate in the body. Particles that are too small are easily excreted by the kidneys or perfuse into systemic circulation, while larger particles are more likely to be recognized and cleared using the mononuclear phagocyte system (MPS) in the liver and spleen. In this study, MSNs with a size of approximately 200 nm were selected, and the particle size distribution before and after MPN modification was measured (Figures 2A,B). The results showed that the particle size of MSNs was primarily distributed below 200 nm, with an average size of 185.9 nm. After MPN modification, the average particle size increased slightly to approximately 200.7 nm, which is attributed to the ∼10 nm thickness of the MPN interfacial layer.

The surface zeta potential of nanoparticles is a critical physicochemical parameter that reflects their charge properties in solution or biological systems. Positively charged nanoparticles tend to bind more readily to negatively charged cell membranes, which facilitates cellular uptake but also leads to rapid clearance by the liver and spleen. Conversely, neutral or negatively charged nanoparticles exhibit longer circulation times in the body. Our results indicate that the surface potential of MSNs was approximately −31.9 mV, which shifted to −25.37 mV after MPN modification (Figure 2C). This change is due to the inherent surface charge of MPN following its interfacial modification on MSNs.

To further confirm the successful introduction of MPN onto MSNs, FT-IR and UV–VIS spectroscopy were used to analyze the chemical composition and functional groups. As shown in Figure 2D, both MPN and MPN@MSN exhibited a broad and strong absorption peak in the 3,000–3,650 cm^-1^ range, corresponding to the characteristic –OH stretching vibrations of epigallocatechin gallate (EGCG). Additionally, peaks at approximately 1,500 cm^-1^, attributed to C–C bonds, and a distinctive Si–O–Si peak in the 1,000–1,200 cm^-1^ range were observed in both MPN and MPN@MSN. A characteristic absorption peak near 500 cm^-1^, corresponding to metal–oxygen (M–O) bonds formed by the coordination of oxygen atoms in the polyphenol ligand with zinc ions, was also detected. These results suggest that EGCG self-assembled on the surface of MSNs via coordination with Zn^2+^ rather than being merely adsorbed.

The UV–VIS spectra showed that MSNs inherently lack significant UV absorption, while MPN-modified MSNs exhibited peaks similar to MPN, with a noticeable blue shift in the absorption maxima (Figure 2E). This shift is attributed to hydrogen bonding and internal molecular interactions, such as charge transfer or steric effects, further confirming the self-assembly of MPN on the MSNs.

SEM images revealed that unmodified MSNs were spherical, with a smooth surface and uniform size of approximately 200 nm. After MPN modification, the particles retained their uniform dispersion but exhibited a rougher surface texture. Elemental mapping analysis (Figure 2F) detected the presence of carbon (C), nitrogen (N), and zinc (Zn) on the surface of MPN@MSN, in addition to silicon (Si) and oxygen (O) from the MSNs. In particular, carbon and nitrogen were derived from EGCG and human serum albumin (HSA), while zinc originated from the coordination complex formed with EGCG. TEM imaging confirmed the hollow porous structure of MSNs, which provides a high potential for drug loading (Figure 2G). After MPN modification, a uniform thin film of approximately 10 nm thickness was observed on the surface of MSNs, indicating successful interfacial modification.

The EGCG molecules contain multiple hydroxyl groups that can form hydrogen bonds with polar amino acid residues in proteins or peptides. Additionally, the hydrophobic aromatic rings in EGCG can interact with hydrophobic regions in proteins or peptides via hydrophobic interactions. These interactions, along with van der Waals forces, π–π stacking, and covalent bonding, enable EGCG to bind to proteins or peptides. During the MPN modification process, specific proteins or antibodies can be introduced to functionalize the nanoparticles, imparting targeting capabilities. In this study, FITC-labeled HSA (FITC-HSA) was used to evaluate the protein-loading capacity of MSNs. The results showed that the fluorescence intensity of MSNs alone was less than 10^2^, which is attributed to their intrinsic fluorescence. After loading with FITC-HSA via MPN, the fluorescence intensity of MPN@MSN significantly increased, with an average value exceeding 10^3^ (Figure 2H). All the results illustrate the ability of MPN to load proteins or peptides onto MSNs effectively.

### 3.2 Drug loading and release

**FIGURE 3:**
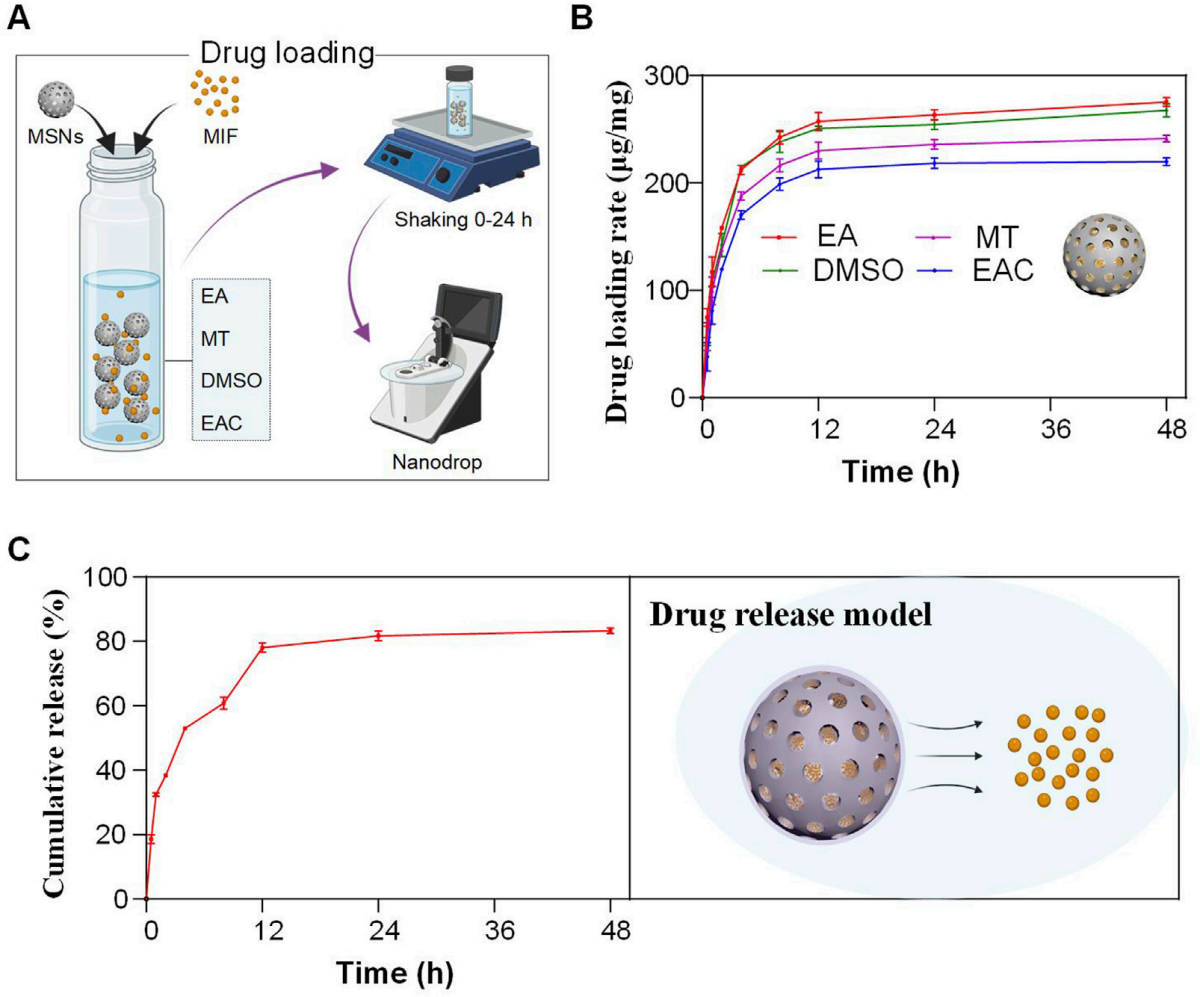
Drug loading and release of MPN@MSN–MIF nanocomposites. **(A)** Schematic workflow of MIF loading into MSNs through solvent-assisted diffusion [EA, ethyl acetate; MT, methanol; DMSO, dimethyl sulfoxide; EAC, ethyl alcohol/chloroform 1:1]. Drug-loading capacity was quantified using NanoDrop spectroscopy at 304 nm. **(B)** Drug loading showed that the loaded MIF in each solvent was essentially at maximum efficiency at 12 h. Inset: Schematic representation of MIF-loaded MSNs. **(C)** Sustained-release profile with cumulative release at different time points (left) and schematic representation of the drug release model (right).

MSNs possess highly ordered mesoporous structures, enabling them to load a large quantity of drug molecules. The drug-loading capacity directly determines the amount of drug that can be transported by the nanoparticles. A higher loading capacity reduces the amount of carrier material required, minimizing potential toxicity and side effects while enhancing therapeutic efficacy. To optimize drug solubility, we evaluated the drug-loading capacity of MSNs in four different solvents: anhydrous ethanol (EA), methanol (MT), DMSO, and ethyl acetate (EAC) (Figure 3A).

The loading profiles (Figure 3B) showed that MSNs reached near-saturation drug loading within 12 h in all solvents, with minimal increases thereafter. The lowest loading capacity was observed in ethyl acetate, with 12-h and 48-h loadings of 213 μg/mg and 220 μg/mg, respectively. In contrast, anhydrous ethanol yielded the highest loading capacity, reaching 257 μg/mg at 12 h and 275 μg/mg at 48 h. Methanol and DMSO exhibited intermediate loading capacities, with 48-h values of 241 μg/mg and 268 μg/mg, respectively. These results indicate that MSNs can achieve high drug-loading capacities in a short time, with anhydrous ethanol being the most efficient solvent.

Subsequently, we encapsulated the drug-loaded MSNs with MPN and evaluated the drug release profiles *in vitro* (Figure 3C). The results showed that the cumulative release reached 78.0% at 12 h and stabilized at 83.3% by 48 h. This sustained-release profile suggests that MPN-encapsulated nanoparticles can maintain effective drug concentrations over time, reducing rapid drug release and potential side effects during systemic circulation. Furthermore, although the sustained drug release cannot be definitively attributed solely to the MPN coating due to insufficient characterization of its structural integrity during the release process, the current results demonstrate that the integrated system achieves an effective sustained-release profile. Further mechanistic investigations remain necessary to elucidate the underlying dynamics.

### 3.3 Biocompatibility of MSNs and MPN@MSN

**FIGURE 4:**
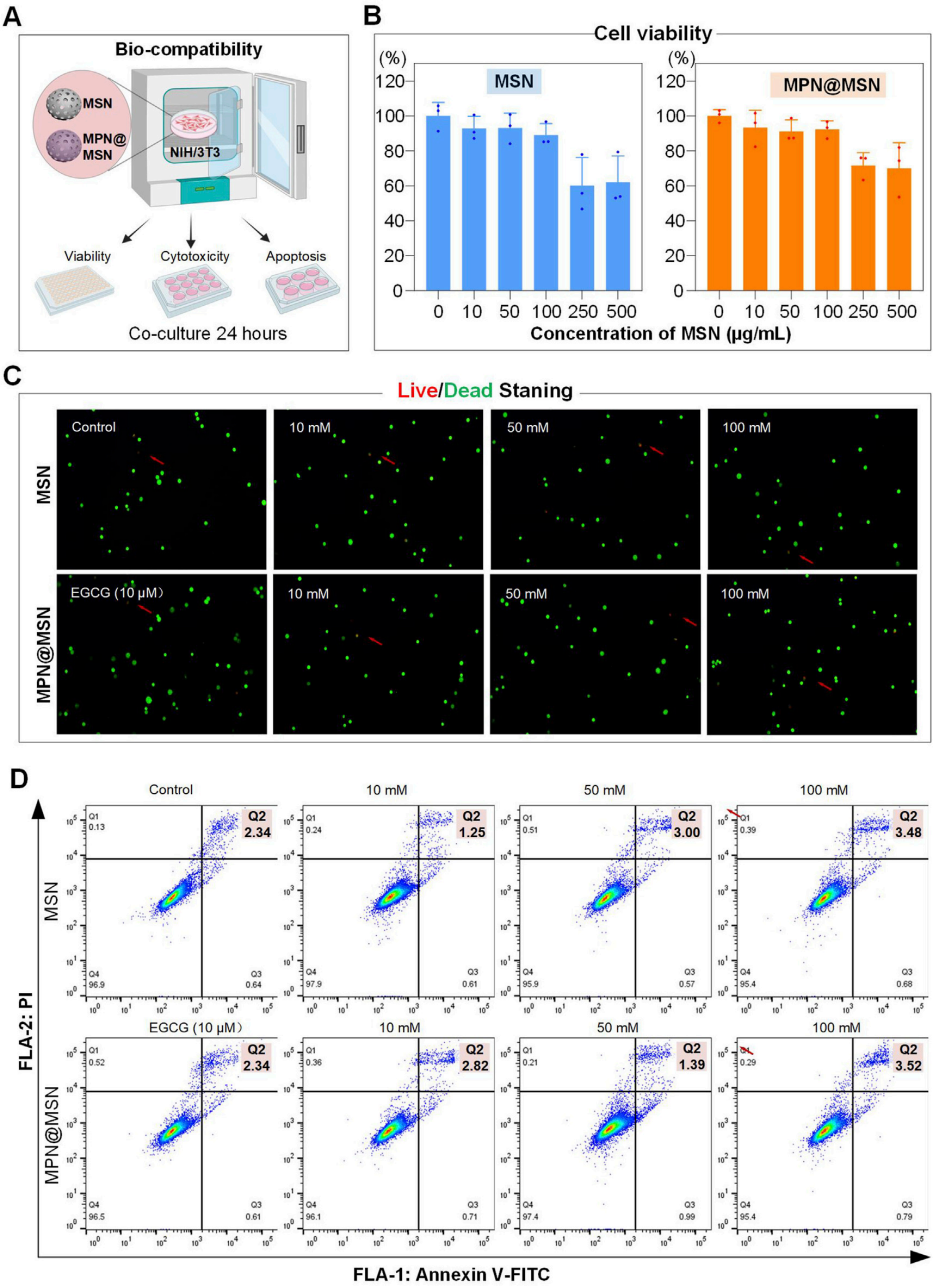
Biocompatibility assessment of MSN and MPN@MSN nanocomposites in NIH/3T3 cells. **(A)** Schematic representation of the co-culture system evaluating viability, cytotoxicity, and apoptosis after 24 h exposure to nanocomposites (0–500 μg/mL). **(B)** Dose-dependent cell viability measured using the CCK-8 assay. **(C)** Live/dead cell fluorescence imaging in NIH/3T3 cells treated with MSN and MPN@MSN nanocomposites. Live cells: green fluorescence; dead cells: red fluorescence (indicated by red arrows). **(D)** Flow cytometry analysis of apoptosis in NIH/3T3 cells treated with MSN and MPN@MSN nanocomposites.

The unique size, surface properties, and composition of nanoparticles can lead to unforeseen biological effects, including toxicity and immune responses. Therefore, thorough evaluation of their biocompatibility is essential for safe clinical applications. In this study, we assessed the biocompatibility of MSNs and MPN@MSN using NIH/3T3 cells as a cell model, focusing on cell viability, cytotoxicity, and apoptosis (Figure 4A).

Cell viability was evaluated across a range of MSN concentrations. The results indicated that cell viability remained above 90% for concentrations up to 100 μg/mL, demonstrating good biocompatibility within this range. However, at higher concentrations (250 μg/mL and 500 μg/mL), cell viability decreased below 80% (Figure 4B). These higher concentrations were not relevant to the intended application and were excluded from further testing. Live/dead cell staining (Figure 4C) and apoptosis assays (Figure 4D) further confirmed that neither MSNs nor MPN@MSN induced significant cell death or apoptosis at concentrations below 100 μg/mL. These findings highlight the excellent biocompatibility of the drug delivery system, making it suitable for *in vivo* applications.

### 3.4 *In vitro* targeting effects and mechanisms

**FIGURE 5:**
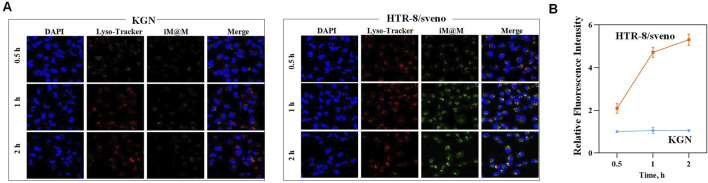
*In vitro* targeting effects of iRGD-mediated MPN@MSNs. **(A)** Time-dependent cellular uptake of iRGD-labeled nanocomposites (green) and lysosomal co-localization (red) in KGN cells (left) and HTR-8/SVneo cells (right). Nuclei counterstained with DAPI (blue). **(B)** Quantitative analysis of relative mean fluorescence intensity in the two cell groups over time (n = 3).

The targeted delivery is based on the well-documented high expression of αvβ3 integrin in trophoblasts, contrasted with its low or absent expression in other reproductive cells such as KGN. This differential expression profile establishes iRGD as a peptide with demonstrated placental tropism, enabling precise guidance of nanoparticles to trophoblast cells (Tang et al., 2024; Greish et al., 2012). We investigated the targeting efficiency of iRGD-mediated MPN@MSNs using human trophoblast-derived HTR-8/SVneo cells and ovarian granulosa KGN cells. To dynamically observe nanoparticle uptake, we co-loaded iRGD and FITC-HSA onto the surface of MSNs and stained cellular lysosomes with a fluorescent probe. The results showed minimal green fluorescence in KGN cells during the 0.5–2-h incubation period, with all fluorescence signals co-localizing with lysosomes (Figures 5A,B). In contrast, HTR-8/SVneo cells exhibited rapid uptake of nanoparticles, with green fluorescence increasing significantly over time and co-localizing with lysosomes (Figures 5A,B). These findings suggest that iRGD significantly enhances the uptake of MPN@MSNs by HTR-8/SVneo cells, leading to higher intracellular drug concentrations in trophoblast cells.

### 3.5 *In vivo* targeting and therapeutic effects

**FIGURE 6:**
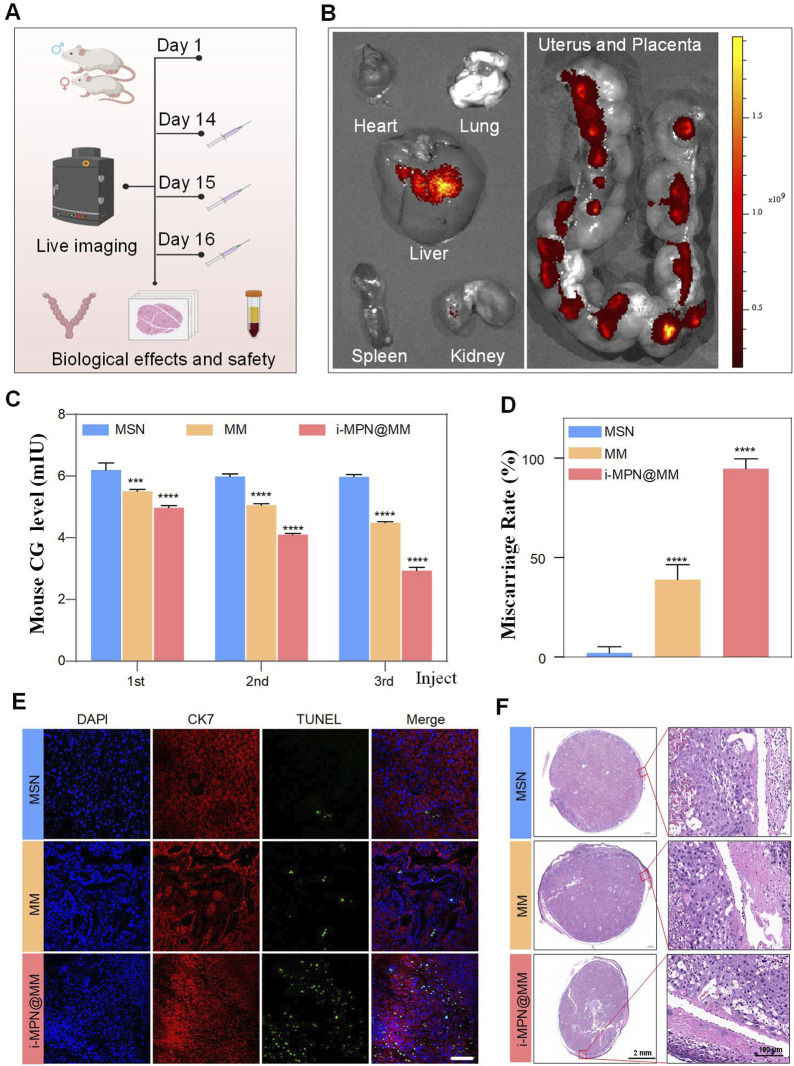
*In vivo* targeting and therapeutic effects of i-MPN@MM. **(A)** Schematic illustration of the nanocomposite’s targeting specificity and therapeutic effects in a mouse model. **(B)** *In vivo* imaging assessment of organ-specific biodistribution for nanocomposites. **(C)** Temporal dynamics of the mCG level in mouse plasma detected through ELISA. For each group, n = 3. **(D)** Miscarriage rate in pregnant mouse models across treatment cohorts. For each group, n = 6. **(E)** Multicolor fluorescence imaging of placental apoptosis in experimental groups. Green, TUNEL-positive apoptotic cells; red, CK7, trophoblast marker; blue, DAPI-stained nuclei. Scale bar: 200 μm. **(F)**.Histological evaluation of mouse placental microarchitecture across treatment groups using H&E staining. Scale bar: 2 mm (left) and 100 μm (right).

The behavior of nanoparticles *in vivo* differs significantly from their *in vitro* performance due to factors such as biological fluid complexity and immune system interactions. To evaluate the *in vivo* performance of iRGD-targeted MPN@MSNs loaded with MIF (i-MPN@MM), we used an ICR pregnant mouse model (Figure 6A). Cy7-labeled nanoparticles were administered to assess their biodistribution and targeting efficiency. Although the pregnant mouse model does not fully replicate the pathological structure of PAS, its placental tissue—particularly trophoblast cells—still exhibits high expression of the iRGD target (αvβ3 integrin). This conserved molecular expression allows the model to effectively demonstrate the intrinsic efficacy of the placental-targeted delivery system itself.

As shown in Figure 6B, i-MPN@MM exhibited significant accumulation in the liver, minimal accumulation in the kidneys, and widespread distribution across the placentas. This pattern is consistent with the liver’s high blood perfusion and its role as the body’s largest metabolic organ, along with the kidneys’ function as primary excretory organs. However, MPN@MM exhibited significant accumulation in the liver and kidneys, and no noticeable retention signal was observed in the placenta (Supplementary Figure S1). The observed placental accumulation demonstrates the ability of i-MPN@MM to deliver MIF, particularly to the placenta, while minimizing distribution to other organs, reducing systemic side effects.

To evaluate therapeutic efficacy, we administered equal doses of PBS, MIF-loaded MSNs (MMs), and i-MPN@MM to pregnant mice on days 14–16 of gestation. Mouse CG levels were measured 24 h after each injection, and the mice were euthanized on day 17 to assess miscarriage rates. CG levels typically decrease in early pregnancy, increase during mid-gestation, and peak in late gestation. Our results (Figure 6C) showed that both MM and i-MPN@MM reduced CG levels, with i-MPN@MM exhibiting a more pronounced decrease. By the third injection, CG levels in the i-MPN@MM group decreased significantly. Furthermore, both groups showed high abortion rates, with i-MPN@MM achieving near-complete abortion (94.71%) (Figure 6D, Supplementary Figure S2).

To investigate the mechanism of abortion, we performed immunofluorescence staining of placental tissues using anti-cytokeratin 7 (CK-7) antibodies to label trophoblast cells, DAPI to stain cell nuclei, and TUNEL to detect apoptotic cells. The results (Figure 6E) showed a significant increase in trophoblast cell apoptosis in both MM and i-MPN@MM groups, with the i-MPN@MM group exhibiting a higher apoptosis rate. H&E staining (Figure 6F) further revealed increased necrotic areas in the placentas of i-MPN@MM-treated mice, consistent with the observed apoptosis. These findings are attributed to the presence of iRGD, which enhances nanoparticle accumulation in trophoblast cells, increasing local drug concentration and inhibiting placental development and implantation.

*In vivo* safety assessments were also conducted. Serum alanine transaminase (ALT) levels, a marker of liver injury, did not differ significantly between treatment groups (Supplementary Figure S3), indicating that liver-targeted nanoparticles did not cause hepatic damage. Hemocompatibility assessment confirmed the intravenous safety of our system, as evidenced by hemolysis assay results demonstrating no significant compromise to erythrocyte integrity (Supplementary Figure S4). Histological examination of major organs (heart, lungs, liver, spleen, and kidneys) revealed no significant structural damage or inflammation in any treatment group (Supplementary Figure S5), further confirming the safety of the drug delivery system.

However, although the pregnant mouse model provides valuable initial insights, it is essential to acknowledge its inherent limitations in fully recapitulating the complex pathophysiology of PAS. These limitations underscore the necessity for future studies to establish and utilize more clinically relevant PAS-specific animal models. Such models are essential for conducting more comprehensive and rigorous validation of the therapeutic efficacy and targeting specificity of the proposed nanoplatform under disease-mimicking conditions.

## 4 Conclusion

In this study, we successfully developed a drug delivery system based on MSNs modified with MPN, offering enhanced drug-loading capacity, controlled release, and effective targeting. The MPN modification was confirmed through various characterization techniques, including FT-IR, UV–VIS spectroscopy, and electron microscopy, which revealed successful integration of polyphenols and metal ions on the MSN surface. This modification significantly improved the surface charge and enabled efficient protein loading, highlighting the versatility of the system for targeted therapeutic applications.

The drug-loading and release profiles established that MPN-modified MSNs are capable of achieving high loading capacities and sustaining drug release over extended periods, reducing the risk of rapid drug elimination and systemic side effects. MPN@MSNs exhibited excellent biocompatibility in both *in vitro* and *in vivo* studies, with negligible cytotoxicity at therapeutic concentrations. Moreover, the iRGD-targeted MPN@MSNs effectively delivered therapeutic agents to trophoblast cells, showcasing their potential for targeted drug delivery under pregnancy-related conditions.

The promising therapeutic outcomes observed in an *in vivo* pregnancy model, along with the high safety profile, underscore the translational potential of MPN@MSNs as an efficient, biocompatible drug delivery platform. These findings lay a foundation for future development of nanomedicines that can offer targeted and controlled release therapies for a range of diseases, particularly those requiring placental or tissue-specific targeting. Further studies are warranted to explore the long-term stability and efficacy of these systems in clinical settings.

## Data Availability

The raw data supporting the conclusions of this article will be made available by the authors, without undue reservation.
