# Supplementary material for: iRGD-mediated MPN functionalization of MSNs for targeted delivery of MIF: a potential strategy for placenta accreta spectrum therapy
Source: Front Bioeng Biotechnol. 2025 Nov 21;13:1684943. doi: 10.3389/fbioe.2025.1684943 (PMC12678236; doi:10.3389/fbioe.2025.1684943)
Supplement: Supplementary file 1 [file Supplementaryfile1.docx]

**Supplementary Information**

**iRGD-Mediated MPN Functionalization of MSNs for Targeted Delivery of MIF: A Potential Strategy for Placenta Accreta Spectrum Therapy**

Fanying Zeng^1,2,3^, Chen Peng ^1,2^, Longxia Tong^1,2,4^ Guolin He^*, 1,2^

^1.^Laboratory of the Key Perinatal Diseases, Key Laboratory of Birth Defects and Related Diseases of Women and Children, Ministry of Education. Chengdu, Sichuan Province, China. 610041.

^2.^Department of Obstetrics and Gynecology, West China Second University Hospital, Sichuan University, Chengdu, Sichuan Province, China. 610041.

^3.^Hi-Tech Zone Hospital for Women and Children, West China Second University Hospital, Sichuan University. Chengdu, Sichuan Province, China. 610041.

^4.^Department of Gynecology and Obstetrics, Meishan Women and Children’S Hospital, Meishan, China. 620500.

**Corresponding Author:** Guolin He, heguolin_19@scu.edu.cn.


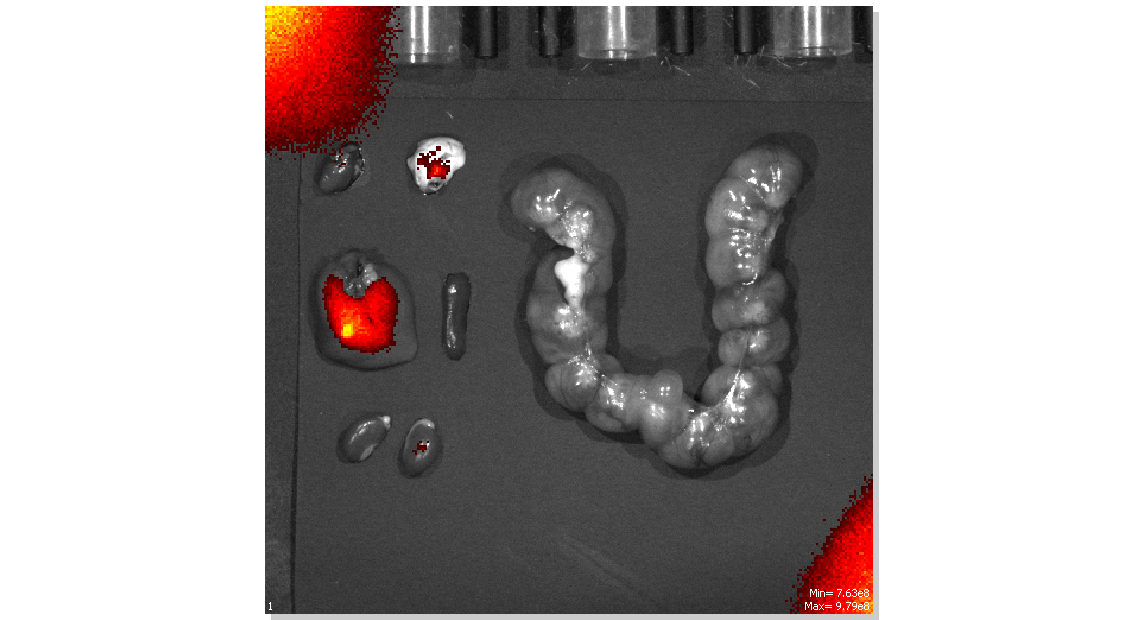


**Supplementary Fig. 1. MPN@MM without iRGD demonstrated significantly higher accumulation in the liver and kidneys, whereas** **no noticeable retention signal was observed in the placenta.**


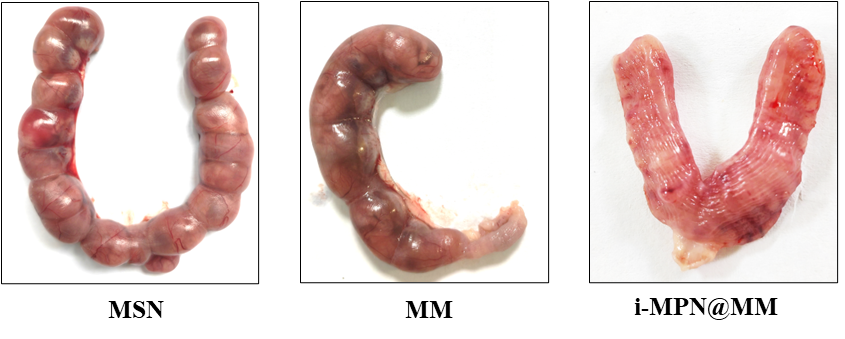


**Supplementary Fig. 2. Representative images of mice uteri from different treatment groups.**

**
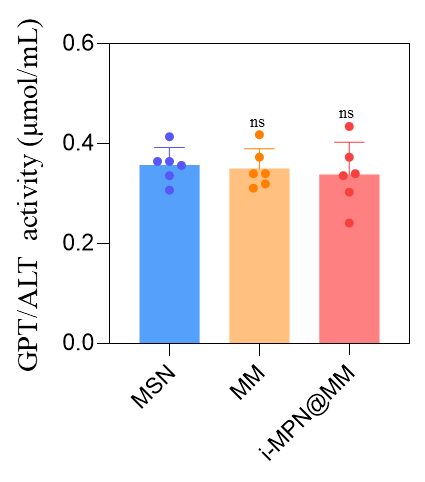
**

**Supplementary Fig. 3. GPT/ALT activity levels in mice from different treatment groups, assessed to evaluate liver safety. Each group n=6.**

**
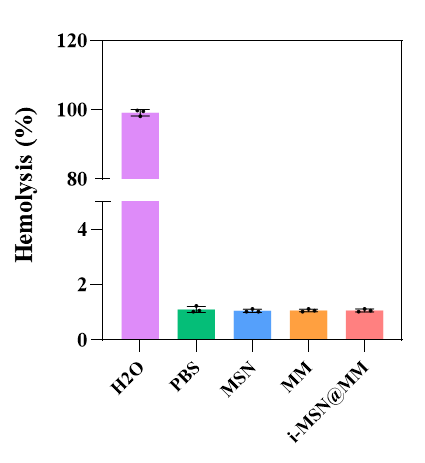
**

**Supplementary Fig. 4. Hemolysis rate results indicate that the drug delivery system has no significant effect on erythrocyte integrity, with H₂O as the positive control and PBS as the negative control.**

**
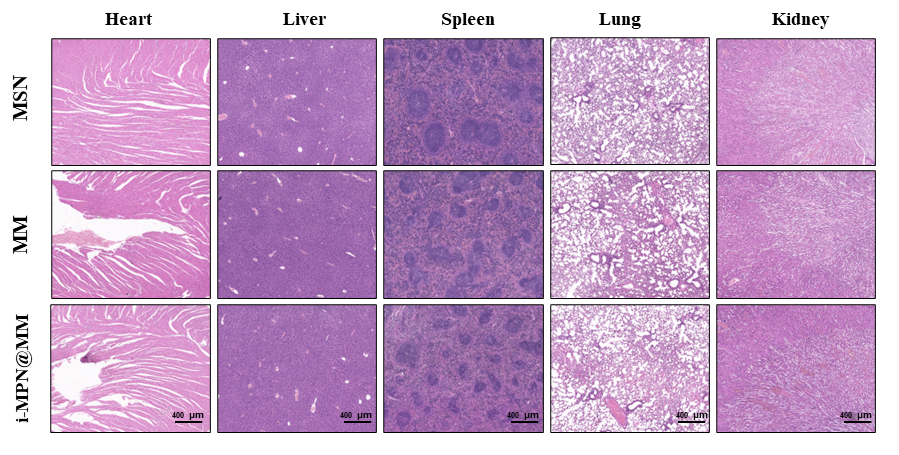
**

**Supplementary Fig. 5. Representative H&E staining images of various organs from mice in different treatment groups. No significant differences in staining results were observed across treatment conditions. Scale bar: 400 μm.**
